# Bone wax as a cause of a foreign body granuloma in a resternotomy : a case report

**DOI:** 10.1186/1749-8090-8-S1-P121

**Published:** 2013-09-11

**Authors:** G Ozerdem, M Hidiroglu, A Kucuker, A Kunt, L Cetin

**Affiliations:** 1 Kayseri Sevgi Hospital, Cardiovascular Surgery Department, Kayseri, Turkey; 2 Ataturk Training and Education Hospital, Ankara, Turkey; 3 Konya Training and Education Hospital, Konya, Turkey

## Background

Bone wax (beeswax) has been used to stop bleeding from the sternal cancellous bone after median sternotomy for many years. Bone wax may sometimes cause complications and surgeons should be aware of this possibility.

## Methods

63 years old woman who had a previous cardiac surgery with median sternotomy was admitted for elective coronary artery bypass surgery. After re-sternotomy, different sized solid masses attached to the sternum and lying on the mediatinal structures consisting of bone wax surrounded by soft granulation tissue was seen. This material extended into the anterior mediastinum. The remnants of bone wax, surrounding inflammatory tissue and adjacent sternal callus were removed. The sternal edges were found to be separated by a cavity containing granular porridge material. The inflammatory tissue was removed together with some of the underlying bone. After performing coronary bypass surgery, the sternotomy was laid open throughout its length and the inflammatory material was scraped out to obtain bleeding bone. No wax was used during this procedure.

## Results

The mass was excised and the postoperative follow up was uneventfull. Histopathologic examination showed foreign-body granulomatous reaction consisting of multinucleated giant cells surrounding wax particles interspersed with abundant mononuclear cells. Bone wax showed a marked foreignbody reaction and lack of bone reformation.

## Conclusion

We report a case in which foreign-body reaction to bone wax applied over open heart surgery required reoperation. Bone wax controls bone bleeding mechanically by occluding the bleeding channels and tamponading the spaces in bone without any biochemical action. The properties and application of bone wax and their possible complications are discussed.

